# Supplementary material for: Structural basis for activation of fluorogenic dyes by an RNA aptamer lacking a G-quadruplex motif
Source: Nat Commun. 2018 Oct 31;9:4542. doi: 10.1038/s41467-018-06942-3 (PMC6208384; doi:10.1038/s41467-018-06942-3)
Supplement: Supplementary file 1 — Supplementary Information [file 41467_2018_6942_MOESM1_ESM.pdf]

Supplementary Information

Structural basis for activation of fluorogenic dyes by an RNA aptamer lacking a G-quadruplex motif

Sandip A. Shelke[1], Yaming Shao[1], Artur Laski[1], Deepak Koirala[1], Benjamin P. Weissman[2], James R. Fuller, Xiaohong Tan[3,4], Tudor P. Constantin[4], Alan S. Waggoner[5,6], Marcel P. Bruchez[4,5,6], Bruce A. Armitage[3,4], Joseph A. Piccirilli[1,2]*

[1]Department of Biochemistry and Molecular Biology, The University of Chicago, Chicago, IL, 60637, USA.

[2]Department of Chemistry, The University of Chicago, Chicago, IL, 60637, USA.

[3]Center for Nucleic Acids Science and Technology, Carnegie Mellon University, Pittsburgh, PA 15213, USA.

[4]Department of Chemistry, Carnegie Mellon University, Pittsburgh, PA 15213, USA.

[5]Department of Biological Sciences, Carnegie Mellon University, Pittsburgh, PA 15213, USA.
[6]Molecular Biosensor and Imaging Center, Carnegie Mellon University, Pittsburgh, PA 15213, USA.

*Corresponding author, email: jpicciri@uchicago.edu

Supplementary Note 1

DIR2s aptamer-Fab BL3-6 interactions:

In the DIR2s-Fab structure, the Fab BL3-6 interacts with its cognate RNA hairpin in a manner similar to that of the previous class I ligase ribozyme and Spinach RNA structures[1, 2] (r.m.s. deviation of = 0.549 Å) involving four complementarity-determining regions (CDRs), L3 from the light chain and H1, H2 and H3 from the heavy chain (Supplementary Fig. 2). In the crystal lattice, which buries a total of 3160 $Å^2$ of surface area, the Fab provides most of the crystal contacts, with a total Fab-RNA buried surface area (BSA) 1440 $Å^2$ and Fab-Fab contacts BSA 1315 $Å^2$. Each Fab interacts with two other symmetry related Fabs mostly through light chain variable domains (Supplementary Fig. 3a, b and 4). Intermolecular RNA-RNA interactions contribute only 405 $Å^2$ of BSA through alternating arrangements of head to head and, end to end stacking interactions (Supplementary Fig. 3c). In the crystal lattice, Fab mediated contacts account for a total of 87% (2755 $Å^2$ out of 3160 $Å^2$) of BSA including the Fab-RNA binding interface within the asymmetric unit (891 $Å^2$). During the crystallization attempts under similar conditions, no crystal formation occurred in the trays setup without Fab, underscoring the importance of the Fab as a crystallization chaperone.

Supplementary Figures

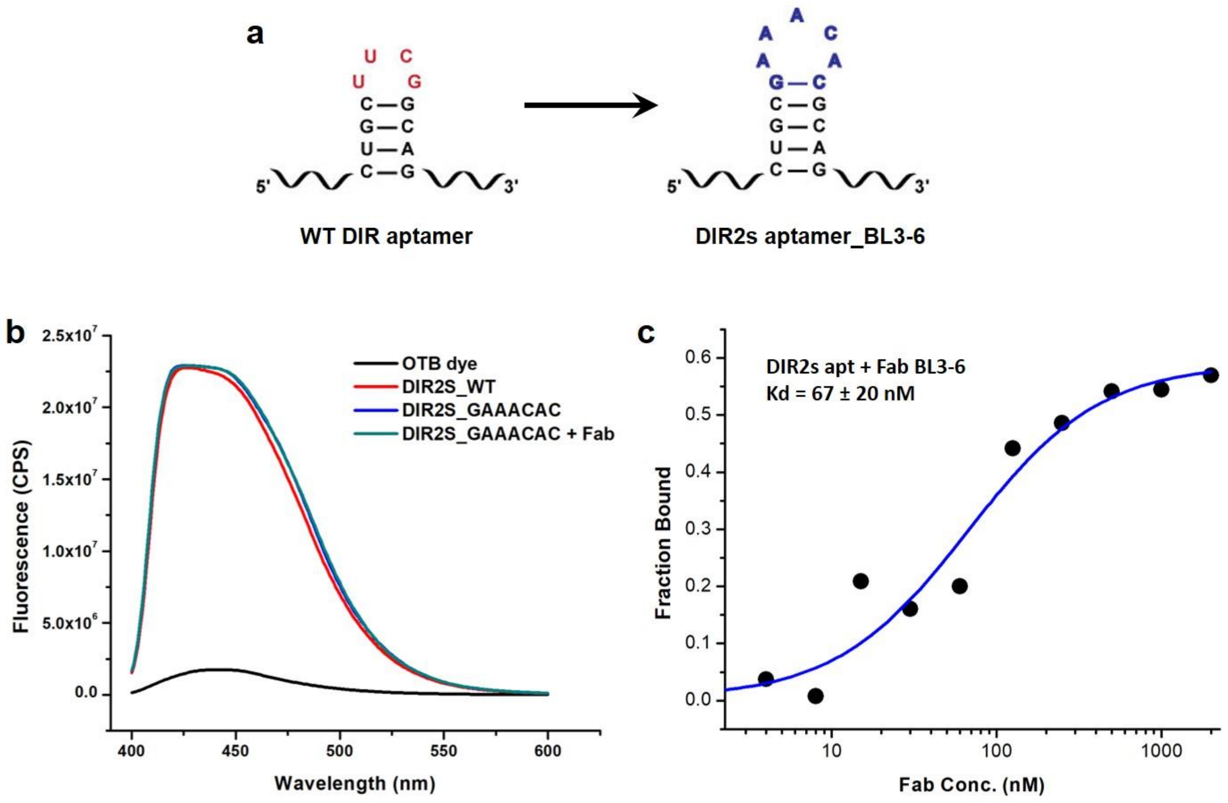

Supplementary Figure 1. Characterization of the DIR2s•Fab BL3-6 complex. (a) Engineering the Fab BL3-6 binding tag into the DIR2s aptamer. The four-nucleotide loop in the wild type (WT) aptamer[3] (shown in red) is replaced with 7-nucleotide (GAAACAC) Fab BL3-6 recognition domain (shown in blue). (b) Fluorescence activation of OTB-SO_3 alone (black), in the presence of DIR2s_WT (UUCG, red), DIR2s_GAAACAC (blue) and DIR2s_GAAACAC in the presence of Fab BL3-6 (green). Excitation wavelength is 380 nm. (c) Binding of Fab BL3-6 to the DIR2s aptamer containing the hairpin graft (GAAACAC) measured by filter binding assay. A fit of the data to the Hill equation gives K_d = 67 ± 20 nM obtained from three consecutive measurements.

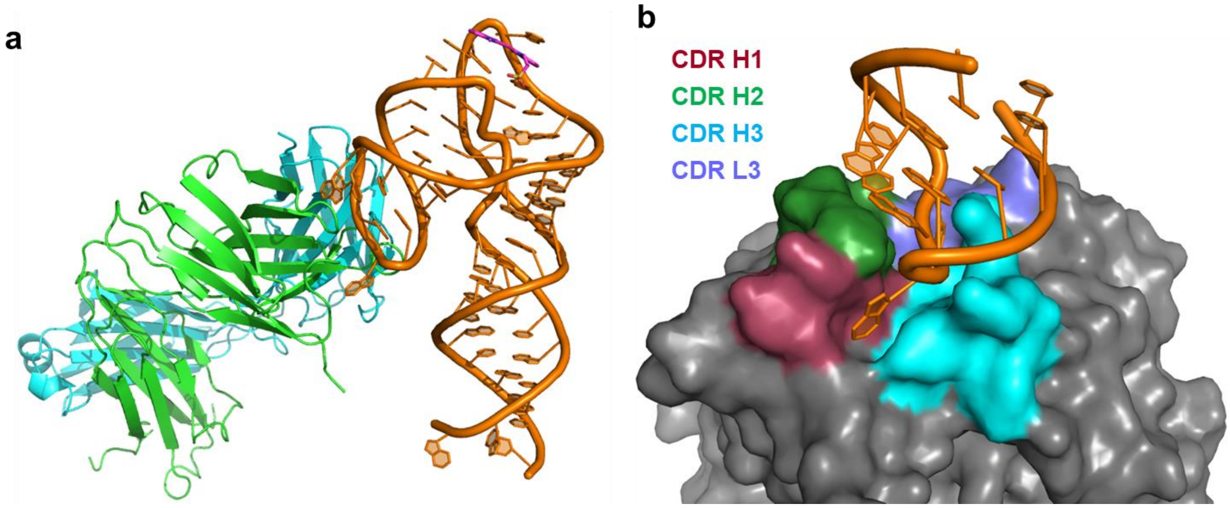

Supplementary Figure 2. The RNA-Fab binding interface. (a) Overall structure of OTB-SO_3 fluorophore bound DIR2s aptamer in complex with Fab BL3-6. (b) The antigenic interface. The stem-loop of the GAAACAC tag (P3-L2, brown cartoon) binds in the groove of Fab BL3-6 created by Fab heavy chain CDRs H1 (maroon), H2 (green), H3 (cyan) and light chain CDR-L3 (purple). CDRs-L1 and -L2 do not make direct contact with the RNA.

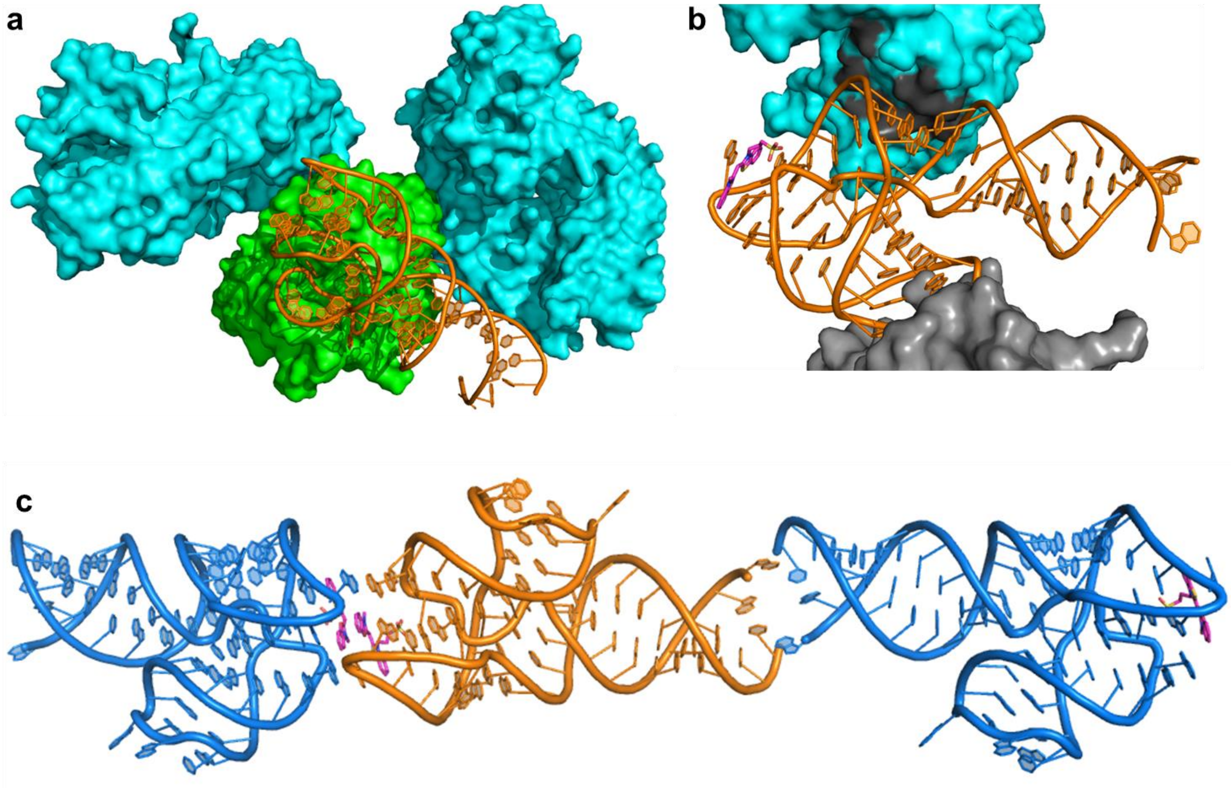

Supplementary Figure 3. The Fab-Fab and RNA-RNA packing in the crystal. (a) Fab-Fab interactions contributing to the lattice packing. One Fab is contacted by two other Fabs (cyan) through light chain interactions. (b) The non-antigenic interfaces engaged by symmetry-related Fab (cyan) in the crystal lattice. Contact residues on the Fabs are highlighted in black and cognate Fab is shown in grey. (c) RNA-RNA contact interfaces in the lattice. One RNA molecule is interacting with two symmetry related RNA molecules in the alternate head to head and tail to tail arrangements.

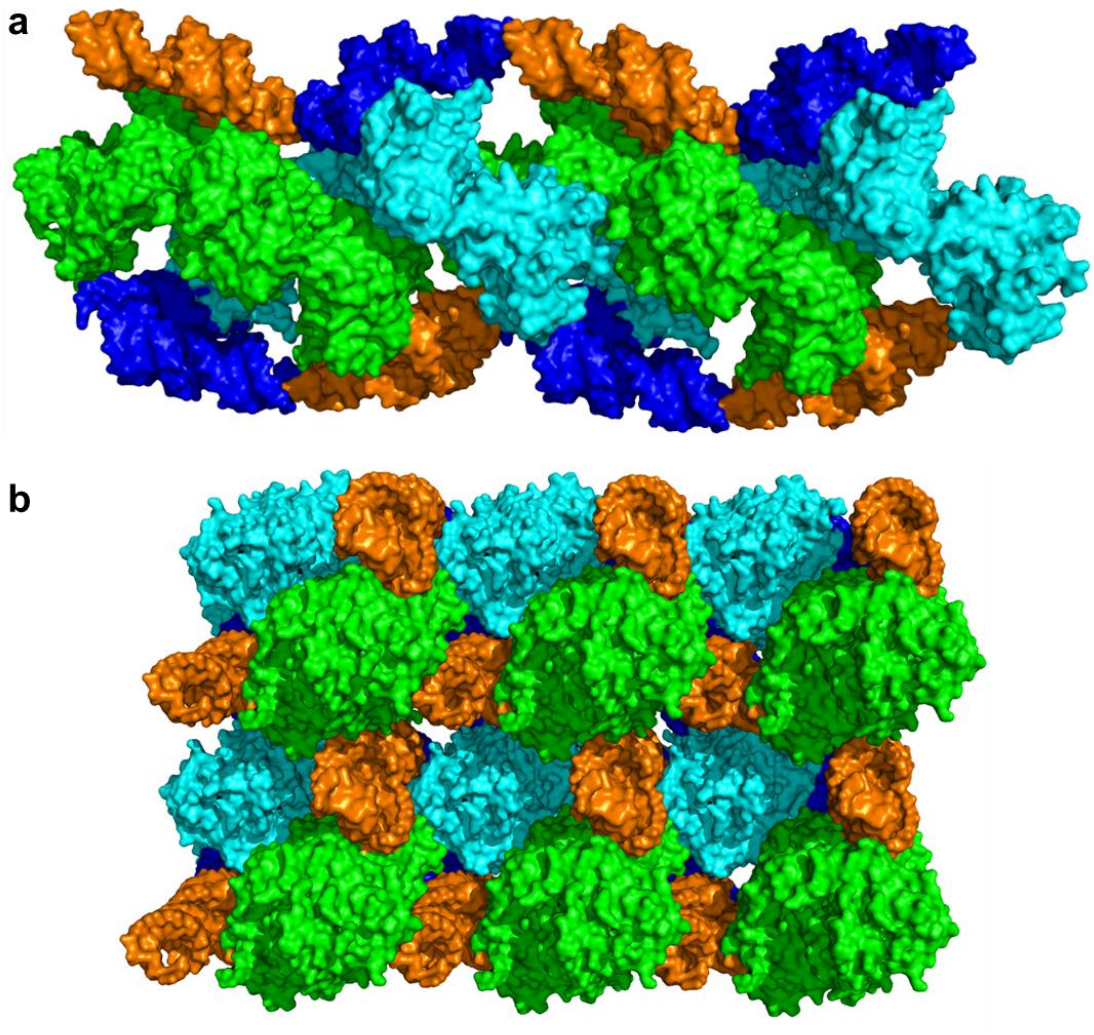

Supplementary Figure 4. Crystal lattice. (a) Crystal packing of the Fab-DIR2s RNA complex (Fab: green, cyan; RNA: brown, blue). In the XY plane, the arrangement of RNA extends via alternating arrangement of head to head and tail to tail interactions. Shown are two layers of RNA in parallel rows. The corresponding Fabs occupy the inner space between the RNA rows and interact with other symmetry-related molecules in a crisscross fashion. (b) The crystal packing in the YZ plane showing tight packing of the Fab-RNA complex. The green and cyan colored Fabs form complexes with the brown and blue RNAs, respectively. Overall, Fab BL3-6 mediated 87% of the total contacts in the crystal packing.

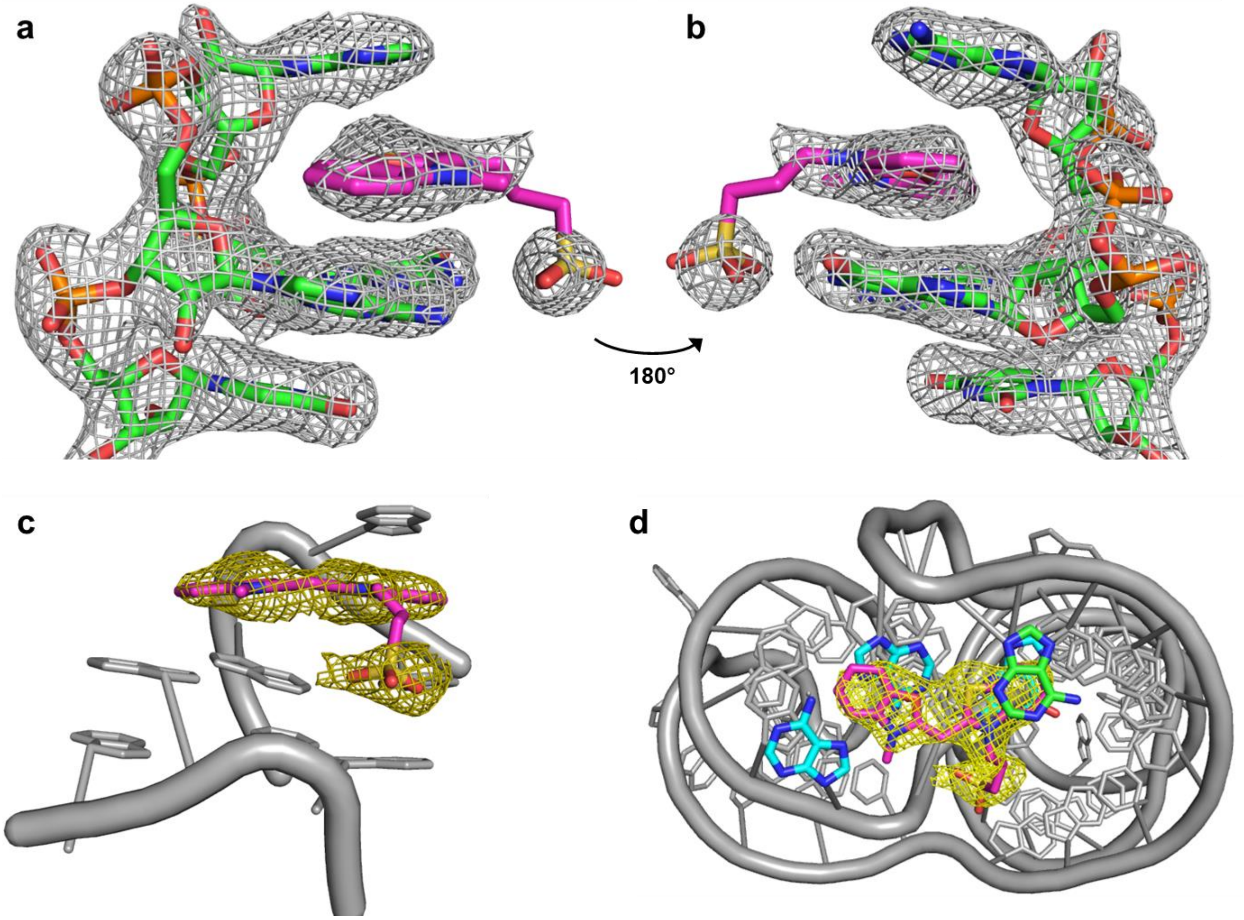

Supplementary Figure 5. DIR2s aptamer binding site bound to OTB-SO_3 ligand with 2|F_o| - |F_c| electron density map. (a) Side view of the ligand binding site in stick representation superimposed on 2|F_o| - |F_c| electron density map. (b) A 180° rotated view of the binding site shown in (a). A clear non-nucleotide density corresponds to the OTB-SO_3 co-planner rings observed. Although no electron density is observed for propyl side chain of OTB-SO_3, a clear density for sulfonate group can be seen. (c) Front view of the binding site. (d) Top view of the aptamer bound to OTB-SO_3 ligand.

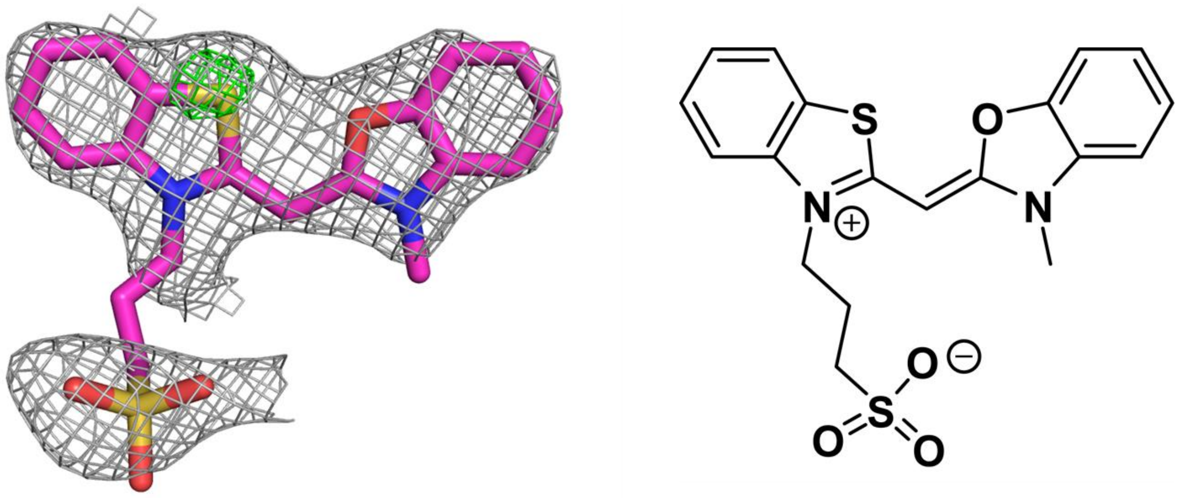

Supplementary Figure 6. Defining the OTB-SO_3 ligand orientation by single wavelength anomalous scattering. To obtain the anomalous difference map, the refined structure was re-refined against the dataset with anomalous signal which was diffracted at wavelength 1.45859 Å. Grey mesh: 2mFo-DFc map contoured at 1σ. Green mesh: anomalous difference map contoured at 3σ. Chemical structure of the OTB-SO_3 is shown on right for reference.

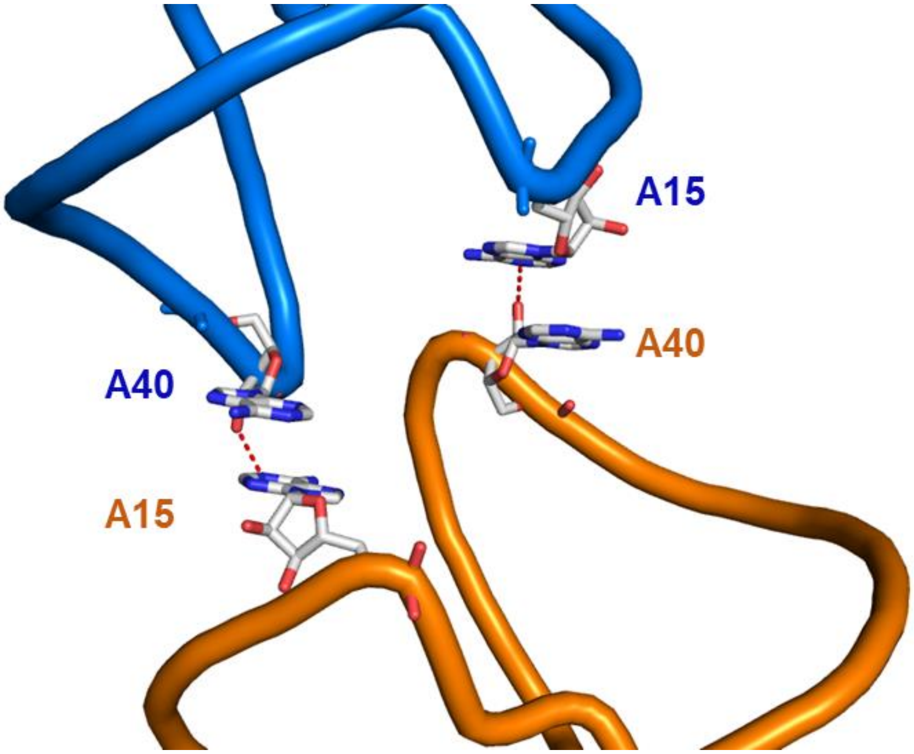

Supplementary Figure 7. RNA-RNA dimer interface. A15 of one RNA (brown) interacts with the A40 of other RNA (blue) in symmetry related molecule and vice-versa through hydrogen bonding and stacking interactions. The two bound OTB-SO_3 and other nucleosides are omitted for clarity.

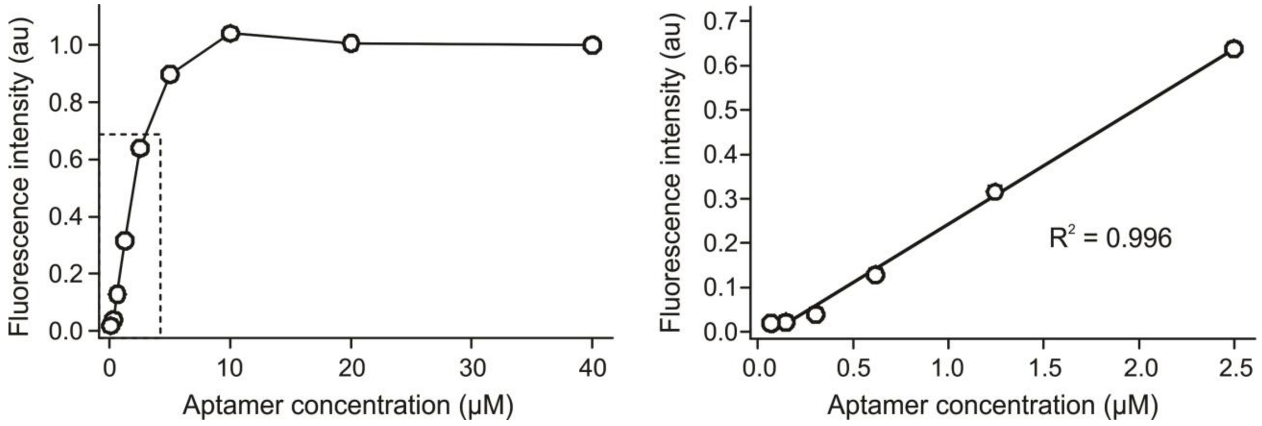

Supplementary Figure 8. Titration of OTB-SO_3 dye with DIR2s RNA aptamer. Left, measured florescence of the dye at constant 10 µM concentration as a function of varying concentrations of the RNA. Plateau occurs at 10 µM of the RNA. Right, fluorescence signal increases linearly (R^2 = 0.996) with aptamer concentration in the range of 0–2.5 µM (dotted box in the left panel). These observations indicate for 1:1 RNA: fluorophore complex formation in solution.

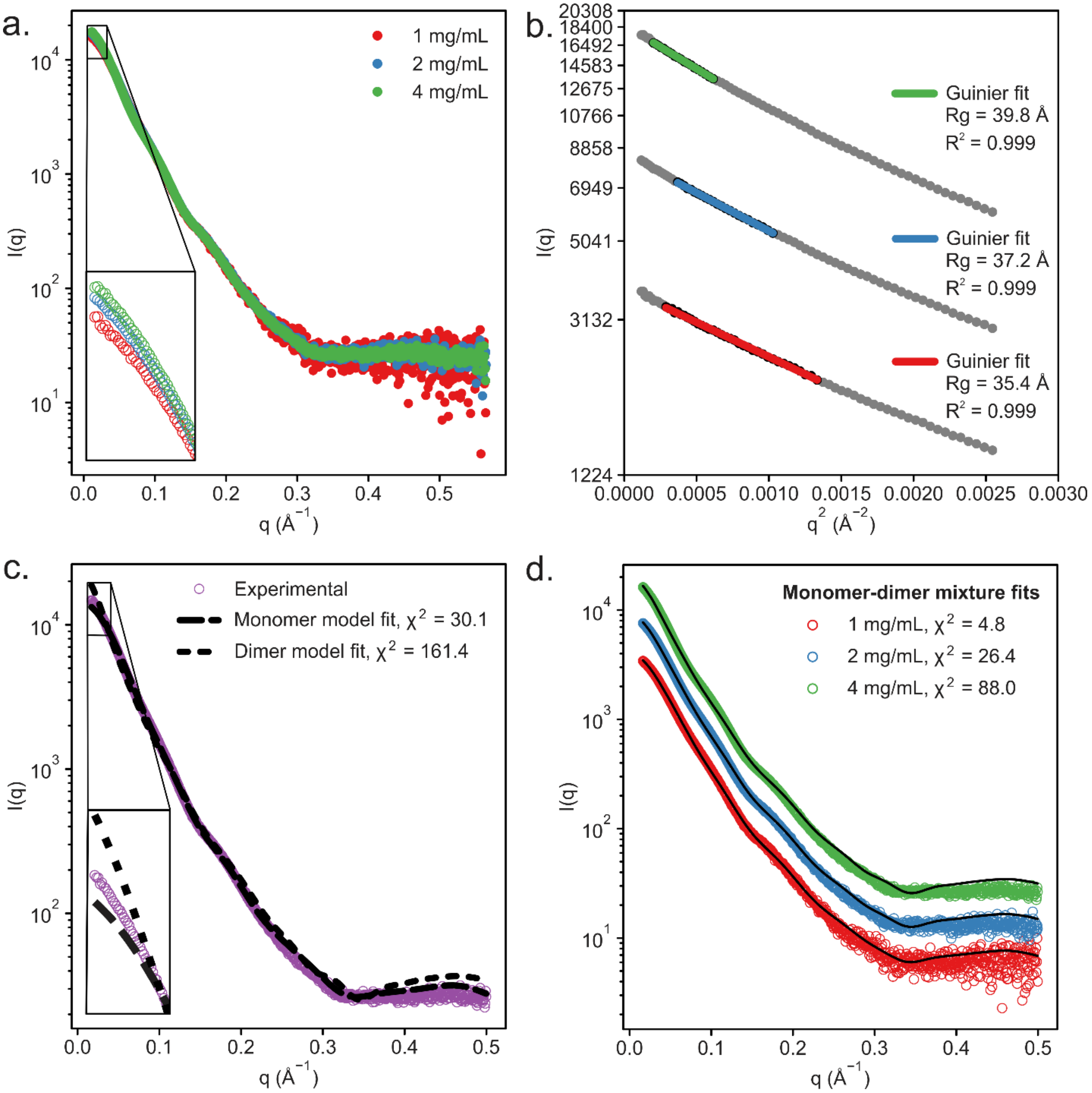

Figure continues on next page

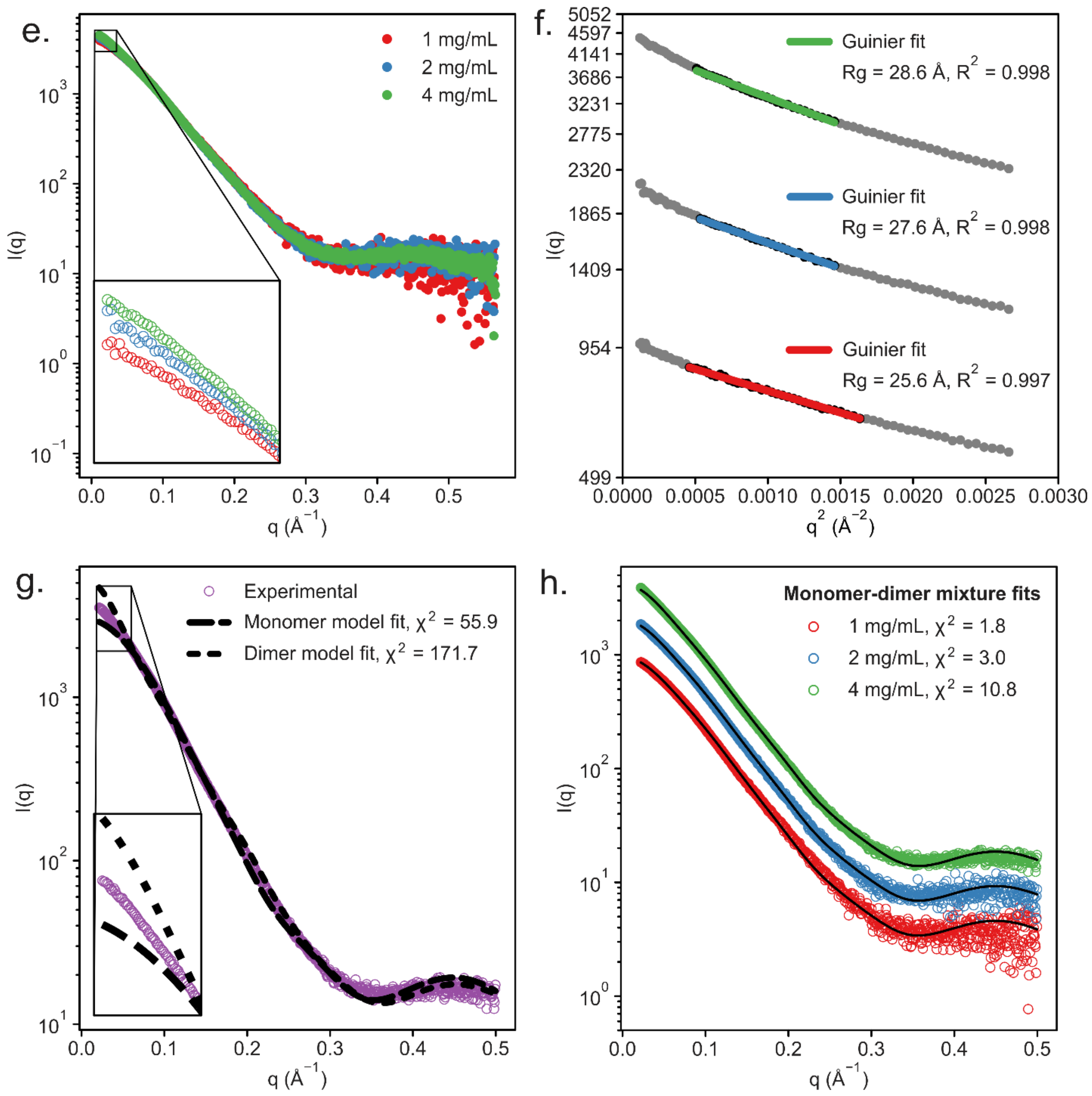

Supplementary Figure 9. SAXS solution phase studies of DIR2s RNA aptamer. For both complexes, RNA + OTB-SO_3 ligand + Fab (a – d) and RNA + OTB-SO_3 ligand (e – h), SAXS datasets from three different concentrations of RNA, 1.0 mg/mL, 2.0 mg/mL and 4.0 mg/mL were collected. (a and e) Scattering profiles for each concentration were scaled to the 4.0 mg/mL intensities. There is good agreement across concentrations except for concentration-dependent variation at low q[4] (zoomed insets in a and e). (b and f) Gunier analysis using AUTORG[5] for each concentration. Concentration-dependent radii of gyration (R_g) within both complexes suggests that

the RNA may have the ability to multimerize in solution. (c and g) A merged curve was generated from the 1 mg/mL and 4 mg/mL datasets, and this was used for fitting against theoretical scattering from monomer and dimer models derived from the crystal structure. In each case (RNA + OTB-SO_3 ligand + Fab and RNA + OTB-SO_3 ligand), a monomer model fits the data better than a dimer model (For RNA + OTB-SO_3 ligand + Fab, χ^2 = 30.1 for monomer model vs. 161.4 for dimer model and for RNA + OTB-SO_3 ligand, χ^2 = 55.9 for monomer model vs. 171.7 for dimer model), but the overall fits are poor, due in large part to deviations at low q (zoomed insets in c and g). (d and h) Better fits to the experimental data at each concentration can be achieved by fitting a variable mixture of crystallographic monomer and dimer. For both complexes and at each concentration, including a minor population of dimer greatly improves the fit at low q and explains the concentration dependence noted above (the fitted fraction of monomer and dimer and the χ^2 for each fit are given in Supplementary Table 1 below). In all cases, the fit predicts ≥ 75% of the particles in solution are monomers. Given that the working biological concentrations of the RNA will be significantly lower than those required for SAXS, and also supported by our other assays (figure 4), we are confident that the fluorescence activity of the RNA aptamer arises from monomers.

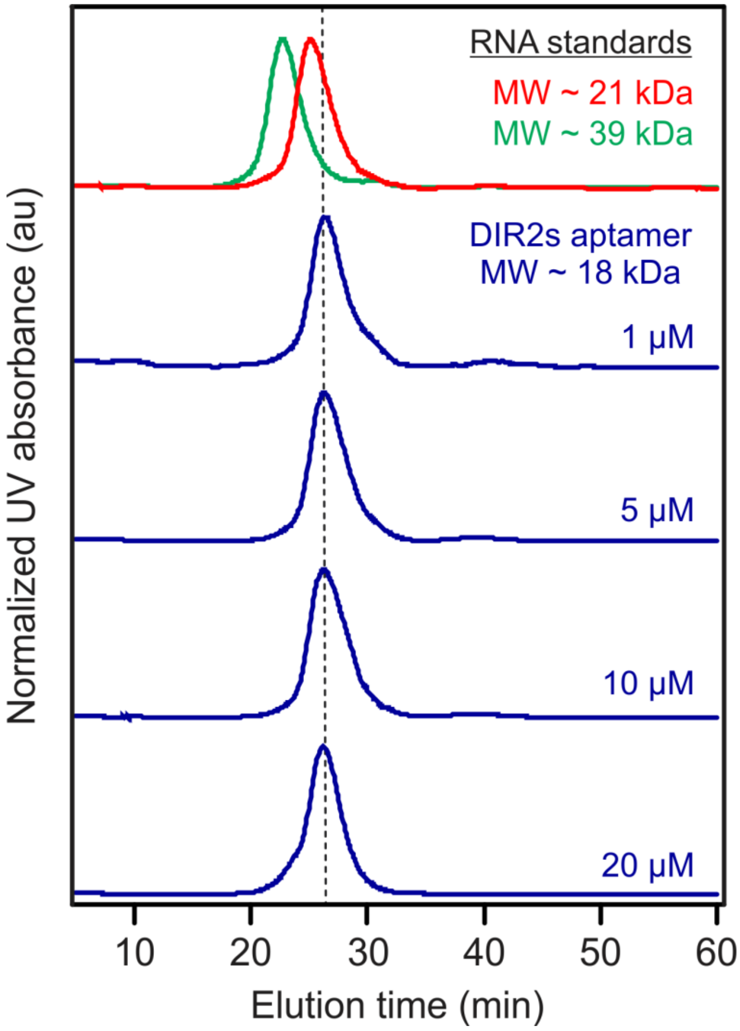

Supplementary Figure 10. Size exclusion chromatography (SEC) of DIR2s aptamer at concentrations ranging from 1 to 20 μM in complex with 2 equivalents of OTB-SO_3 dye for each concentration. RNA standards with molecular weight (MW) ~21 kDa (Red) and ~39 kDa (green) serve as the references for the monomeric (~18 kDa) and dimeric (~36 kDa) forms of the aptamer, respectively. Over the concentration range tested, DIR2s RNA elutes as a single peak slightly slower than 21 kDa reference (see vertical dotted line for guidance) expected for the monomeric form of the aptamer in the solution. All SEC experiments were carried out in a 50 mM Tris pH 7.4, 150 mM NaCl, 5 mM $MgCl_2$ buffer at 4 °C using a Superdex 200 10/300 GL column (GE healthcare).

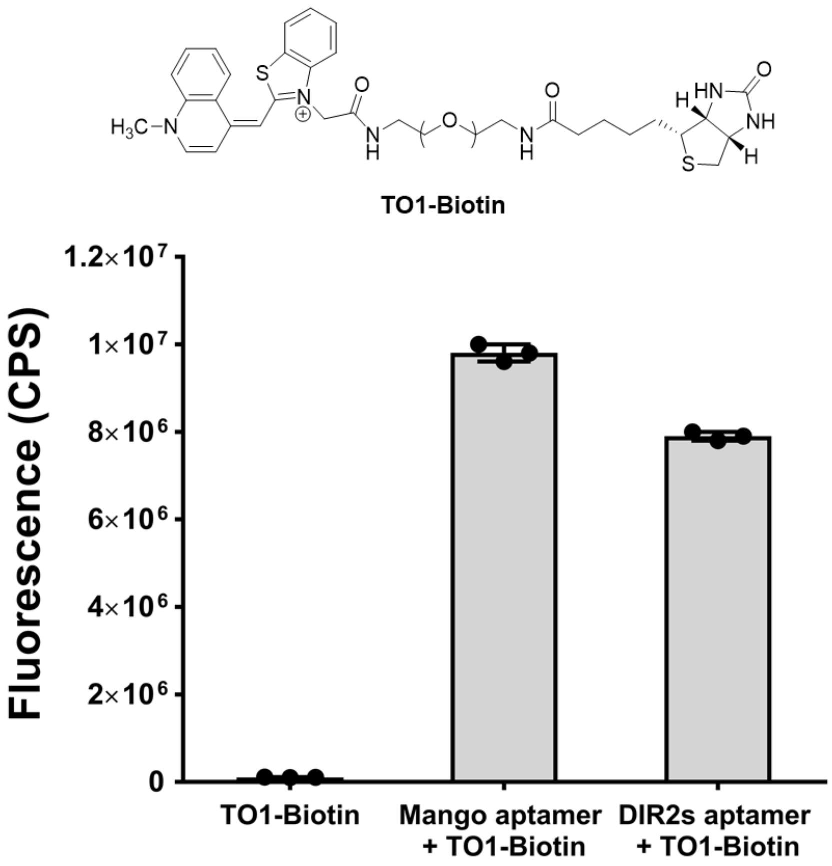

Supplementary Figure 11. Chemical structure of TO1-Biotin (top) and florescence enhancement of TO1-Biotin dye by its cognate (RNA Mango) and DIR2s RNA aptamers. The λ_{max} for emission spectrum for TO1-Biotin only, TO1-Biotin with mango aptamer and TO1-Biotin with DIR2s aptamer were 535 nm, 544 nm and 539 nm, respectively. The error bars indicate mean and standard deviations from three consecutive measurements.

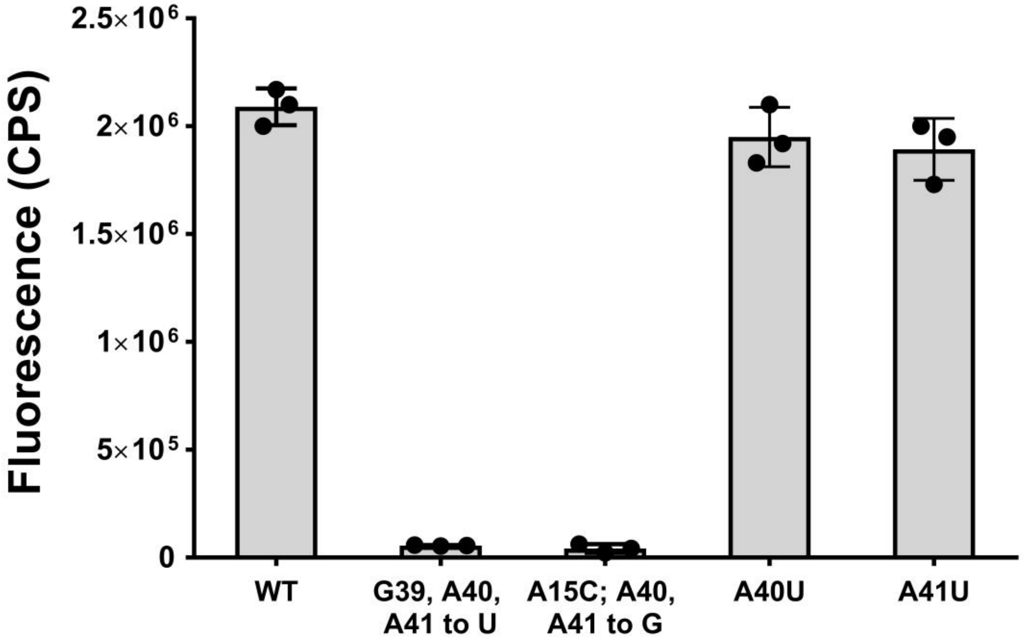

Supplementary Figure 12. Important nucleobases at the binding site and the P1 stem that contribute for DIR-SO_3 binding and fluorescence activation. RNA and DIR-SO_3 concentrations were 3 µM and 6 µM, respectively. The error bars indicate mean and standard deviations from three consecutive measurements.

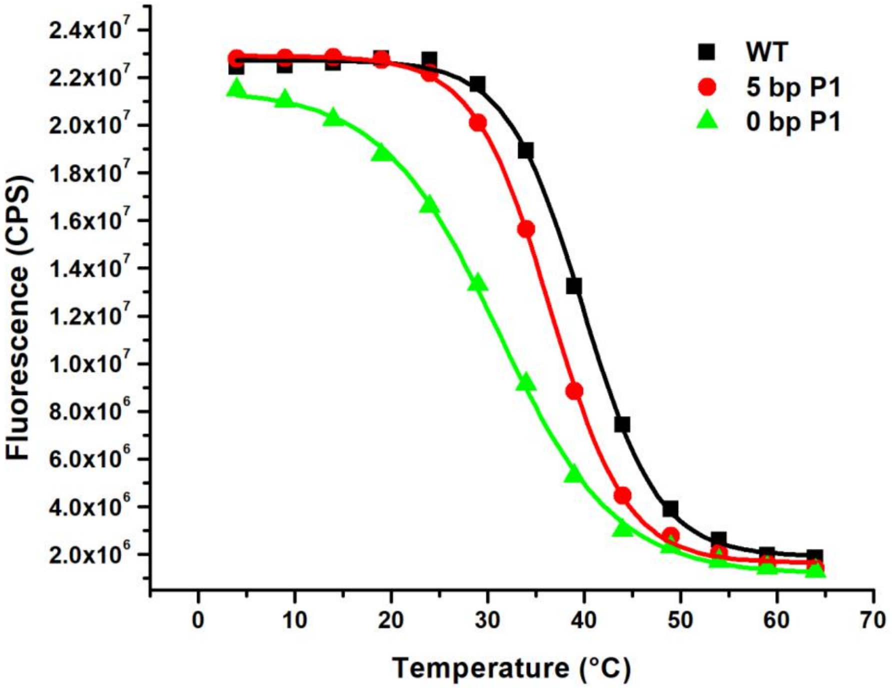

Supplementary Figure 13. Thermal melting curves monitored by fluorescence measurement for DIR2s aptamer and its truncated variants with saturating OTB-SO_3 dye (excitation and emission wavelengths 380 nm and 426 nm, respectively). (black) wild type DIR2s aptamer (red) DIR2s aptamer with five base-pair P1 stem and (green) DIR2s aptamer with zero base-pair P1 stem. RNA 3 μM, OTB-SO_3 6 μM in in 50 mM Tris pH 7.4, 150 mM NaCl, 5 mM $MgCl_2$. The estimated transition temperature for the WT aptamer is 40 °C and for the five- and zero-base-pair stem variants it is 37 °C and 31 °C, respectively.

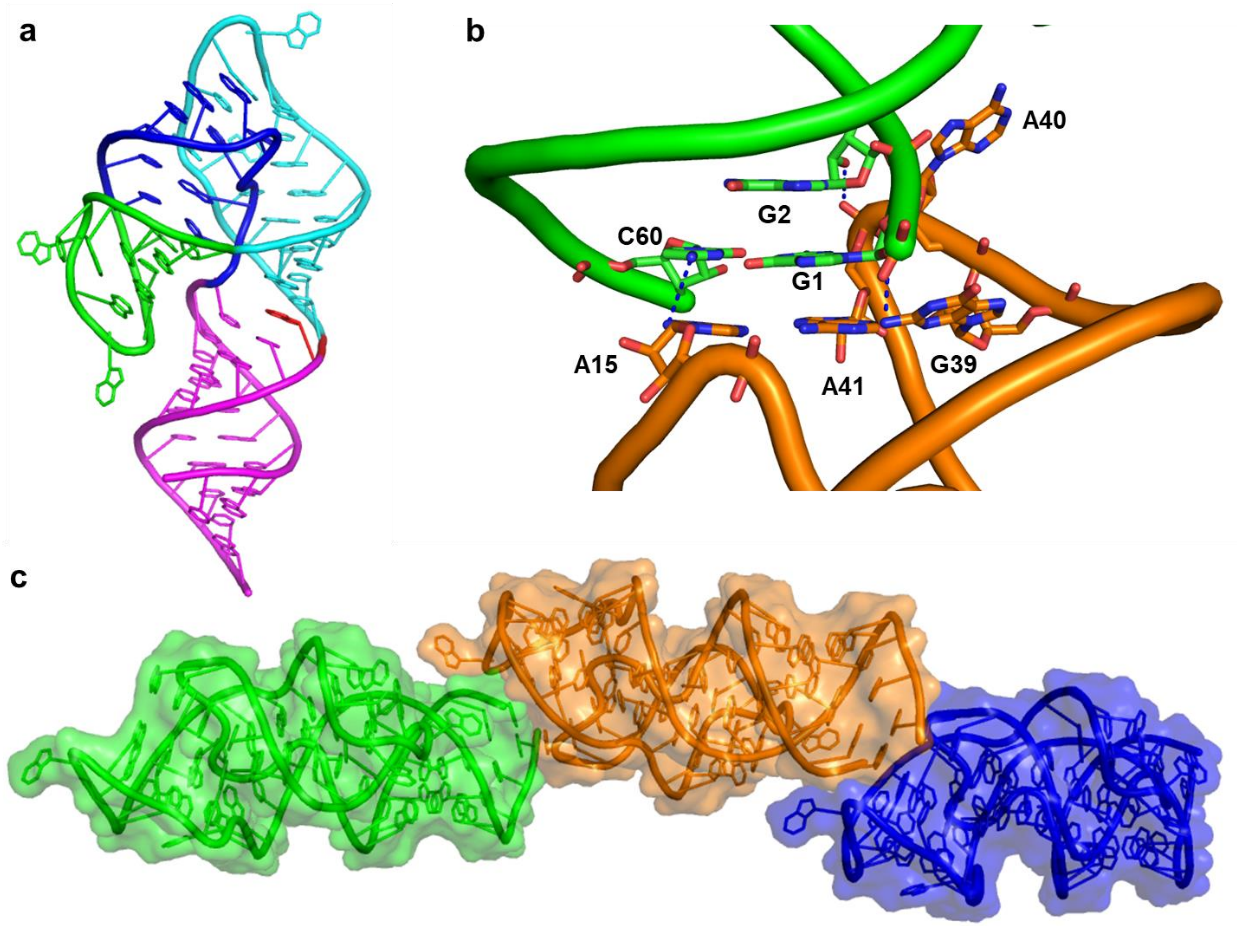

Supplementary Figure 14. Apo form of DIR2s aptamer. (a) Overall structure. Fab is not shown for clarity. (b) RNA-RNA interactions with symmetry related molecules in the crystal packing. The terminal base-pair (G1-C60) of one RNA stacks on the apex of the loop-loop interface from another RNA. Specifically, G1 and C60 stack on A41 and A15, respectively. O4’ of G1 accepts a hydrogen bond from the exocyclic amino group of G39, and C60 donates a hydrogen bond via its exocyclic amine to N3 of A15. The 2’-OH of G2 donates a hydrogen bond to the non-bridging oxygen of A41. (c) RNA-RNA contacts in crystal packing.

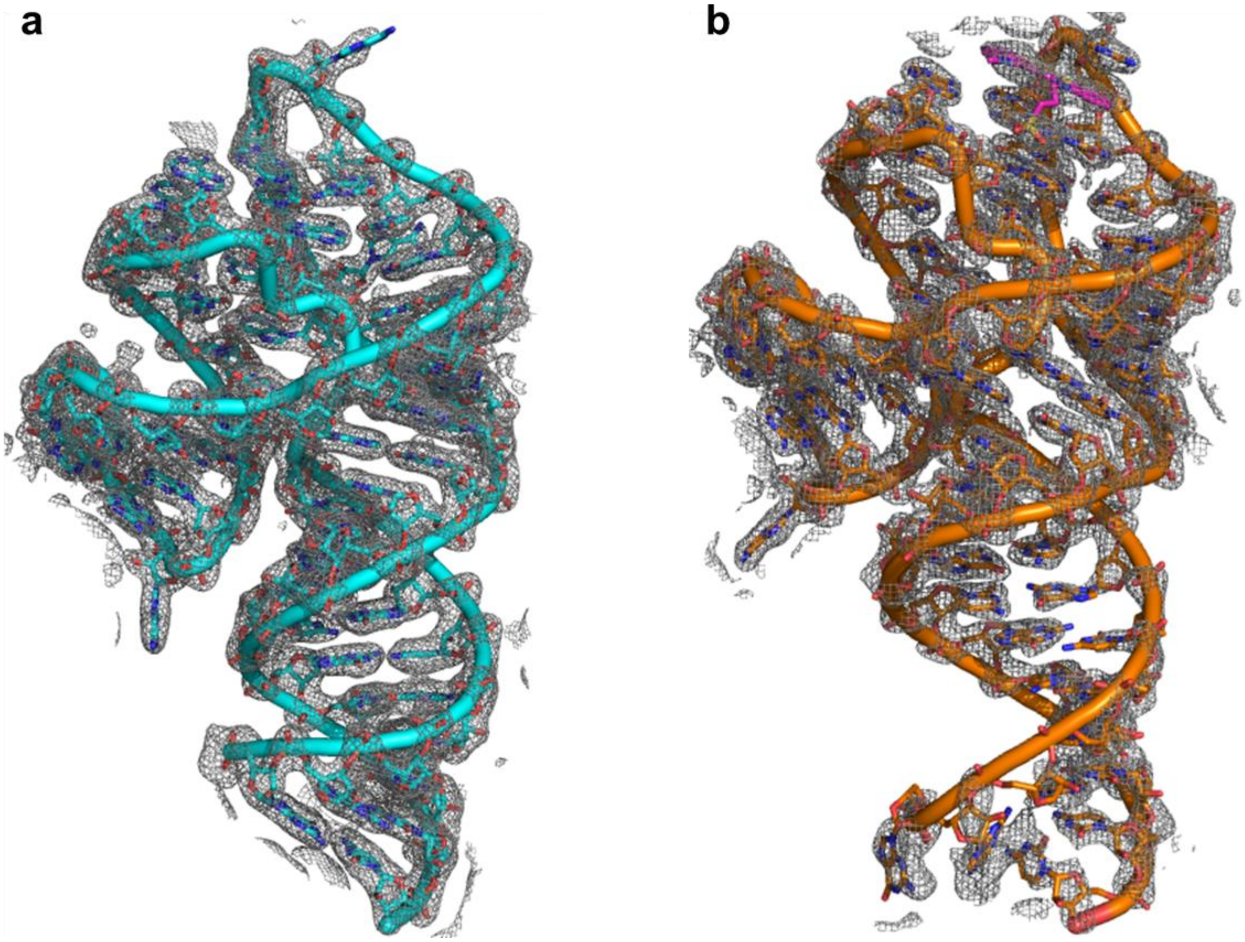

Supplementary Figure 15. The overall $|F_o|$ - $|F_c|$ electron density maps of the DIR2s aptamer apo form (a) and OTB-SO_3 bound form (b).

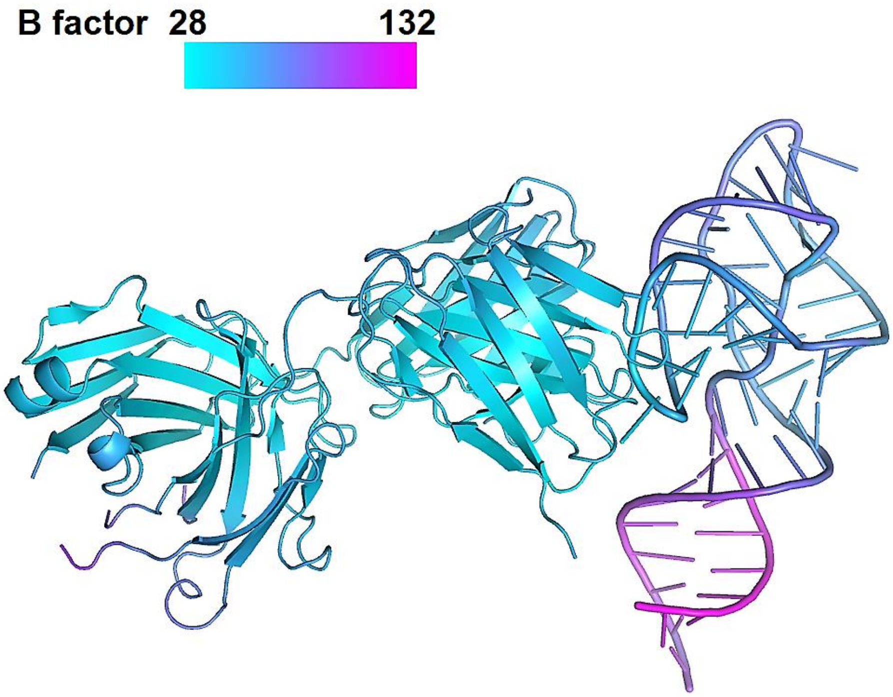

Supplementary Figure 16. A cartoon representation of the structure Fab●DIR2s●OTB-SO_3 complex colored according to B-factors of each region. OTB-SO_3 dye has been omitted for clarity.

Supplementary Tables

Supplementary Table 1. Summary of SAXS data collection, processing and SAXS-derived parameters for both RNA + OTB-SO_3 + Fab and RNA + OTB-SO_3 samples.

**Data collection**	
Instrument	SIBYLS beamline<br>Advanced Light Source (ALS)<br>Berkeley, CA
Beam geometry	Superbend Synchrotron Light<br>100 x 100 micron
Wave length (Å)	1.27
Q range ($Å^{-1}$)	0.012 – 0.54
Exposure time / slicing time (s)	10 / 0.3
Temperature (K)	283
Sample concentrations (mg/mL)	1, 2 and 4

**Software**	
Cross-concentration merging	ALMERGE[6]
Model intensity calculation and single model fitting	FOXS[7]
Model mixture fitting	OLIGOMER[8]
Guinier analysis	AUTORG[5]

Radii of gyration from Guinier analysis:

Sample	R_g (Å) for RNA + OTB-SO_3	R_g (Å) for RNA + OTB-SO_3 + Fab
1 mg/mL	25.6 ± 1.5	35.4 ± 0.8
2 mg/mL	27.6 ± 2.0	37.2 ± 0.7
4 mg/mL	28.6 ± 2.0	39.8 ± 0.9
Crystal structure monomer	19.3	31.0
Crystal structure dimer	32.5	56.4

Fitted monomer – dimer fractions and χ^2:

Sample	Concentration	Monomer fraction	Dimer fraction	χ^2
RNA + OTB-SO_3	1 mg/mL	0.84	0.16	1.8
	2 mg/mL	0.81	0.19	3.0
	4 mg/mL	0.79	0.21	10.8
RNA + OTB-SO_3 + Fab	1 mg/mL	0.88	0.12	4.8
	2 mg/mL	0.81	0.19	26.4
	4 mg/mL	0.74	0.26	88.0

Supplementary Table 2. DNA and RNA sequences used in this study.

**Description**	**Sequence**
DIR2s RNA	5’GGAUGCGCCUUGAAAAGCCUGCUUCGGCAGCUGGUGAAUGACAGCUAUGGCGCAUCC 3’
DIR2s DNA template	5’*gcgtaatacgactcactata*GGATGCGCCTTGAAAAGCCTGCTTCGGCAGCTGGTGAATGACAGCTATGGCGCATCC 3’
DIR2s_GAAACAC RNA	5’GGAUGCGCCUUGAAAAGCCUGC<u>GAAACAC</u>GCAGCUGGUGAAUGACAGCUAUGGCGCAUCC 3’
DIR2s_GAAACAC DNA template	5’*gcgtaatacgactcactata*GGATGCGCCTTGAAAAGCCTGCGAAACACGCAGCTGGTGAATGACAGCTATGGCGCATCC 3’
G39, A40, A41 to U	5’GGAUGCGCCUUGAAAAGCCUGCGAAACACGCAGCUGGUUUUUGACAGCUAUGGCGCAUCC 3’
A15C; A40, A41 to G	5’GGAUGCGCCUUGAACAGCCUGCGAAACACGCAGCUGGUGGGUGACAGCUAUGGCGCAUCC 3’
A40-A-A41	5’GGAUGCGCCUUGAAAAGCCUGCGAAACACGCAGCUGGUGAAAUGACAGCUAUGGCGCAUCC 3’
5 bp P1	5’GGCCUUGAAAAGCCUGCGAAACACGCAGCUGGUGAAUGACAGCUAUGGCC 3’
0 bp P1	5’GGAAAAGCCCGCGAAACACGCAGCUGGUGAAUGACAGCU 3’
A40U	5’GGAUGCGCCUUGAAAAGCCUGCGAAACACGCAGCUGGUGUAUGACAGCUAUGGCGCAUCC 3’
A41U	5’GGAUGCGCCUUGAAAAGCCUGCGAAACACGCAGCUGGUGAUUGACAGCUAUGGCGCAUCC 3’
Abasic G39	5’GGAUGCGCCUUGAAAAGCCUGCGAAACACGCAGCUGGU__AAUGACAGCUAUGGCGCAUCC 3’
Abasic A40	5’GGAUGCGCCUUGAAAAGCCUGCGAAACACGCAGCUGGUG__AUGACAGCUAUGGCGCAUCC 3’
Acceptor	5’ GGAUGCGCCUUGAAAAGCCUGCGAAACACGCAG 3’
Donor (WT)	5’ CUGGUGAAUGACAGCUAUGGCGCAUCC 3’
Donor (abasic G39)	5’ CUGGU__AAUGACAGCUAUGGCGCAUCC 3’
Donor (abasic A40)	5’ CUGGUG__AUGACAGCUAUGGCGCAUCC 3’
DNA Splint	5’ GCTGTCATTCACCAGCTGCGTGTTTCG 3’
Forward Primer	5’GCGTAATACGACTCACTATAGGATGCG 3'
Reverse Primer	5' GmGmATGCGCCATAGCTGTCATTC 3'
5 bp P1 reverse primer	5' GGCCATAGCTGTCATTCACC 3'
0 bp P1 reverse primer	5' AGCTGTCATTCACCAGCTGC 3'

Lowercase letters indicate T7 promoter sequence; underline nucleotides indicate Fab BL3-6 binding site and __ indicates abasic site.

Supplementary References:

1. Koldobskaya Y, *et al.* A portable RNA sequence whose recognition by a synthetic antibody facilitates structural determination. *Nat. Struct. Mol. Biol.* **18**, 100-106 (2011).

2. Huang H, *et al.* A G-quadruplex–containing RNA activates fluorescence in a GFP-like fluorophore. *Nat. Chem. Biol.* **10**, 686-691 (2014).

3. Tan X, Constantin TP, Sloane KL, Waggoner AS, Bruchez MP, Armitage BA. Fluoromodules Consisting of a Promiscuous RNA Aptamer and Red or Blue Fluorogenic Cyanine Dyes: Selection, Characterization, and Bioimaging. *J. Am. Chem. Soc.* **139**, 9001-9009 (2017).

4. Rambo RP, Tainer JA. Accurate assessment of mass, models and resolution by small-angle scattering. *Nature* **496**, 477 (2013).

5. Petoukhov MV, Konarev PV, Kikhney AG, Svergun DI. ATSAS 2.1–towards automated and web-supported small-angle scattering data analysis. *J. Appl. Crystallogr.* **40**, s223-s228 (2007).

6. Franke D, Kikhney AG, Svergun DI. Automated acquisition and analysis of small angle X-ray scattering data. *Nuclear Instruments and Methods in Physics Research Section A: Accelerators, Spectrometers, Detectors and Associated Equipment* **689**, 52-59 (2012).

7. Schneidman-Duhovny D, Hammel M, Tainer JA, Sali A. Accurate SAXS profile computation and its assessment by contrast variation experiments. *Biophys. J.* **105**, 962-974 (2013).

8. Konarev PV, Volkov VV, Sokolova AV, Koch MH, Svergun DI. PRIMUS: a Windows PC-based system for small-angle scattering data analysis. *J. Appl. Crystallogr.* **36**, 1277-1282 (2003).
